# Revisiting the Risk Factors for Endometriosis: A Machine Learning Approach

**DOI:** 10.3390/jpm12071114

**Published:** 2022-07-07

**Authors:** Ido Blass, Tali Sahar, Adi Shraibman, Dan Ofer, Nadav Rappoport, Michal Linial

**Affiliations:** 1 The Rachel and Selim Benin School of Computer Science and Engineering, The Hebrew University of Jerusalem, Jerusalem 91904, Israel; ido.blass@mail.huji.ac.il; 2 Alan Edwards Pain Management Unit, McGill University Health Centre, Montreal, QC H3G 1A4, Canada; talisahar@gmail.com; 3 Department of Computer Science, The Academic College of Tel Aviv-Yaffo, Tel Aviv 69978, Israel; adi.shribman@gmail.com; 4 Department of Software and Information Systems Engineering, Faculty of Engineering Sciences, Ben-Gurion University of the Negev, Be’er Sheva 84105, Israel; dan.ofer@mail.huji.ac.il; 5 Department of Biological Chemistry, Institute of Life Sciences, The Hebrew University of Jerusalem, Jerusalem 91904, Israel; nadavrap@bgu.ac.il

**Keywords:** machine learning, UK-Biobank, pelvic pain, women’s health, CatBoost, features engineering

## Abstract

Endometriosis is a condition characterized by implants of endometrial tissues into extrauterine sites, mostly within the pelvic peritoneum. The prevalence of endometriosis is under-diagnosed and is estimated to account for 5–10% of all women of reproductive age. The goal of this study was to develop a model for endometriosis based on the UK-biobank (UKB) and re-assess the contribution of known risk factors to endometriosis. We partitioned the data into those diagnosed with endometriosis (5924; ICD-10: N80) and a control group (142,723). We included over 1000 variables from the UKB covering personal information about female health, lifestyle, self-reported data, genetic variants, and medical history prior to endometriosis diagnosis. We applied machine learning algorithms to train an endometriosis prediction model. The optimal prediction was achieved with the gradient boosting algorithms of CatBoost for the data-combined model with an area under the ROC curve (ROC-AUC) of 0.81. The same results were obtained for women from a mixed ethnicity population of the UKB (7112; ICD-10: N80). We discovered that, prior to being diagnosed with endometriosis, affected women had significantly more ICD-10 diagnoses than the average unaffected woman. We used SHAP, an explainable AI tool, to estimate the marginal impact of a feature, given all other features. The informative features ranked by SHAP values included irritable bowel syndrome (IBS) and the length of the menstrual cycle. We conclude that the rich population-based retrospective data from the UKB are valuable for developing unified machine learning endometriosis models despite the limitations of missing data, noisy medical input, and participant age. The informative features of the model may improve clinical utility for endometriosis diagnosis.

## 1. Introduction

Endometriosis is an estrogen-dependent, chronic gynecological disorder that is defined by the presence of endometrial-like tissue outside the uterus, primarily in the pelvic tissues and organs [1]. The endometrial-like implants elicit an inflammatory response [2] that involves angiogenesis, fibrosis, and sensory neuron innervation [3]. The most common symptoms include severe pelvic pain, dysmenorrhea, dyspareunia, other chronic pain conditions, fatigue, and infertility [4,5]. Most cases occur in women from menarche to menopause.

Endometriosis affects an estimated 5% to 10% of reproductive-age women, but many remain undiagnosed or are misdiagnosed [6,7]. As a consequence of improved diagnostic tools and increased awareness, reports on endometriosis have increased [8,9], yet the variability in endometriosis prevalence estimates remains high [10]. The diagnosis process for women in the USA and UK reported about 25 years ago showed that, on average, it took more than 10 years between the onset of reported pain symptoms and surgical diagnosis [11,12]. Even now, depending on medical and social awareness, it may take 4–11 years from the emergence of the first symptom to a diagnosis [13,14]. The gold standard for diagnostics is laparoscopic surgery. Non-invasive diagnostic methods (e.g., ultrasonography, MRI) have improved and are increasingly applied. However, providing a correct diagnosis is still challenging due to a shortage of specialists and trained physicians [15]. Surgical techniques for lesion removal may temporarily reduce some of the symptoms and are applied to increase the chances of a natural conception [16]. Nevertheless, the recurrence of lesions following surgery occurs in 5–25% of cases [17]. A variety of hormonal medications are used to suppress endometrial growth, such as contraceptive pills, GnRH agonists, and recently, a GnRH antagonist [18]. These can be used to relieve endometriosis symptoms, support the diagnosis of endometriosis, or prevent recurrence after surgery [19]. Endometriosis symptoms have a substantial impact on the physical and emotional well-being of young women [20]. Prior to diagnosis, women spend time and money, consume unnecessary drugs, and often go through excessive medical procedures.

Along with the increase in awareness and emphasis on women’s health in the last few decades, medical health records and epidemiological data were used to find risk factors for endometriosis [21]. Studies identified several factors that were consistently associated with an increased risk of endometriosis. The most common risk factors in the literature are prolonged estrogen exposure from early menarche to late menopause and a shorter menstrual cycle length. Furthermore, early adult BMI is inversely related to endometriosis [22]. Other factors, such as increased height and low birth weight, were shown to be risk factors in some but not all studies. Notably, smoking has been shown in some studies to increase and in others to decrease the risk of endometriosis. Inconsistency is often associated with lifestyle variables (e.g., alcohol use) [21,23]. The impact of dietary products on endometriosis risk may represent confounding factors that are prone to ongoing changes in lifestyle [24]. However, none of these factors have been found to be explicitly and conclusively used for the diagnosis of endometriosis. When the surgically diagnosed group was compared to a matched group examined by pelvic MRI, fertility history was found to be a major risk in both groups [25].

Twin and family studies support a genetic component to endometriosis [26] and family association studies confirm it to be a complex inherited trait. Women with first-degree relatives with endometriosis were found to be at a higher risk of the disease, compared to those with unaffected relatives [27]. The estimated heritability is 0.47–0.51 based on twin studies, and 0.26 according to common SNP-based heritability [28]. Several genome-wide association studies (GWAS) have identified several association to single-nucleotide polymorphisms (SNPs) with a low effect size [29,30]. Still, over a dozen genetic loci associated with hormonal regulation pathways [28] and an immune-inflammation signature [31] were proposed. GWAS-identified loci seem to explain a small fraction of the variability and are mostly associated with the severe forms of the condition. Currently, the power of genetic-based diagnosis is too low to be useful.

At present, no blood biomarker provides sufficient diagnostic accuracy, according to a Cochrane systematic review that covered 141 studies and 122 proposed blood biomarkers (a total of 15,141 participants) [32]. While advances in non-invasive tests, including imaging and miRNA profiles, carry promising diagnostic potential, the clinical recommendations still lag behind [16].

The goal of the current study was to assess the predictive power of an expanded list of variables related to endometriosis using the UK-Biobank (UKB) cohort and machine learning-based models. The richness and coherence in data collection and data recruitment allowed us to minimize selection bias and test the relative contribution of a very large number of factors simultaneously, while overcoming the challenge of missing data. The UKB also provides individual-level data with the associated genetics, therefore allowing us to include personalized genetics into a combined predictive model. In this study, we combined time-sensitive clinical data (e.g., ICD-10 medical diagnoses), information associated with nutrition and lifestyle (e.g., dairy preference), and genetic data (i.e., GWAS common variants) via a machine learning model. The performance of the gradient boosting predictive model approach in view of alternative machine learning methods, and the clinical utility of personalized medicine are discussed, as are the most impactful features.

## 2. Methods

### 2.1. UKB Data Extraction and Processing

The UK Biobank (UKB) is a population-based database with detailed medical, genotyping, and lifestyle information on 500,000 people between the ages of 40 and 69 at the time of recruitment [33]. UKB recruited the participants during 2006–2010 from across the UK. All analyses were based on the 2019 UKB release. We further removed genetic relatives by keeping only one representative of each kinship group of related individuals. This resulted in a dataset with 145,671 participants. Disease classification is based on clinical information encoded by ICD-10 codes. We used the main or secondary diagnosis (UKB data-fields 41,202 and 41,204, respectively) with the age of the diagnosis.

We addressed each data field according to the missing information included. In some cases, the information was only relevant to a subset of the studied population. For example, the ‘age of the first episode of depression’ is only valid for those who replied positively to ‘ever felt depression’. Among those subjects, 80% had not reported on the age of their first episode of depression. In other cases, the fraction of missing data was restricted to the absence of measurements of participants that did not know the answer (e.g., breastfed as a baby). Supplementary Figure S1 sorts a set of variables by their fraction of missing information. In cases where multiple values were reported for a specific field (e.g., BMI from repeated visits), only the last value was considered. Data fields that were found related to endometriosis by the literature (and consulting with clinicians) were collected, along with all of the participants’ documented ICD-10 code diagnoses.

A protocol for age-dependent matching of the endo group and control group was implemented by performing a stochastic matching process between the two groups. The objective of this protocol is to keep the majority of the samples while matching the year of birth distribution. In practice, we randomly chose 71,088 samples from the group of women without endometriosis diagnosis (control group) with a similar birth year distribution as the birth year distribution of women with an endometriosis diagnosis (endo group). The rest of the analysis was performed on the matched set. See Supplementary Text S1 for the pseudocode used.

### 2.2. Genetic Analysis

The UKB released genotyped data for all participants. The genotyping scheme is based on 805,426 preselected genetic variations. Based on the imputation protocol, the number of variants was expanded to about 9 M variants that passed quality control [34]. We used the Open Targets (OT) platform to select currently available knowledge on endometriosis genetics [35]. OT is a public database that unifies evidence for drugs, their targets, and their associations with human diseases. We used the genetic platform that compiled the top-scored variants from GWAS summary statistics as extracted from the GWAS catalog [36]. The OT genetic association scores were applied to extract an informative list of variants associated with endometriosis. We gathered an unfiltered list of 189 SNPs linked to 221 genes from OT (based on OT quality criteria, some genes lack associated SNPs). We extracted the SNPs associated with endometriosis as reported by OT. A total of 65 unique genetic variants were used in our model (Supplementary Table S2). A more elaborate set of associated variants for endometriosis was used to model the genetics (total 399 variants, Supplementary Table S2). This extended list was extracted from the unified cohort from the UKB and Ireland [37]. We focused on Caucasian women by limiting the analysis to participants who self-reported themselves as British, Irish, or other “white” background [codes 1, 1001, 1002, 1003, respectively, in Ethnic background, UKB data field 21000] and were classified as Caucasians based on their genetic ancestry (Genetic ethnic group, data-field 22006). We further performed a set of analyses for the mixed ethnicity group (*n* = 178,438 women).

### 2.3. Machine Learning Methodology

We tested several models, including Random Forest, Logistic Regression, and Linear Discriminant Analysis, and compared their performance. We also applied CatBoost, which belongs to a family of tree-based gradient boosting algorithms that perform well in big data with missing data [38,39]. The CatBoost model was trained for 1000 iterations using early stopping on a separate held-out validation subset. In each step of the algorithm, a decision tree-based learner is created, using the previous iterations’ decision tree residuals as a gradient for minimizing the current tree’s loss function. For each iteration, CatBoost uses a random permutation of the training set. The subset is used in order to build the decision tree and to build target statistics for the categorical features by mapping these features into a continuous space [40]. We trained the following three types of models according to the type of data used: (a) Attributes and measurements that were compiled from the reported risk factors for endometriosis in the literature and other fields that were proposed by medical experts (Supplementary Table S1). (b) Medical diagnoses, as indexed by ICD-10 codes. (c) Genetic variants based on endometriosis GWAS from marker SNPs, and an expanded list used to construct endometriosis-PRS (polygenic risk score; Supplementary Table S2) [37]. We used the receiver operating characteristic area under the curve (ROC-AUC) as the evaluation metric. We used SHAP (SHapley Additive exPlanations) to estimate the features’ importance [39]. SHAP values provide a numerical estimate of the marginal impact of a feature, given all other features.

### 2.4. Feature Engineering

In addition to the UKB data fields, we engineered features which were not explicitly found in the UKB. Estrogen exposure, for example, was calculated by reducing the age of menarche from the age of menopause. Many of the features from the ICD-10 diagnosis fields were extracted from the UKB and converted prior to their use in the predictive model (Supplementary Table S3). From the reported dates of any diagnosis available in the UKB, we calculated the age when the participant was diagnosed for each of the ICD-10 records available for that person. The feature of the amount of ICD-10 diagnoses was calculated by summing up the diagnoses available in the medical record that were accumulated prior to the endometriosis diagnosis age. In this case, for the control group, a matching protocol was performed in order to determine the age threshold for such counting.

### 2.5. Statistical Tests

We applied a post hoc univariable analysis using the Kruskal–Wallis test for continuous variables and Pearson’s chi-squared test for binary variables. For each feature, we calculated the standardized mean difference (SMD) as its summary statistics. The SMD expresses the size of the effect relative to the variability observed. Formally, we measured the mean outcome between endometriosis patients and the control group relative to the standard deviation of the outcome among control participants. The univariable analysis was limited to Q1–Q3 to improve statistical robustness.

## 3. Results

### 3.1. Unification of Data from UKB: Case-Control Population-Based Groups

The primary goal of this study was to review current risk-factor knowledge and evaluate its contribution to endometriosis prediction. To this end, we systematically collected a set of phenotypes and measurements extracted from the UKB database. As a population-based resource, the UKB is based on standardized data collection protocols. The UKB includes over 500,000 participants collected from 23 medical centers across the UK, who were recruited over the years 2006–2010 for participants aged 40–69 (54.4% are females). The average age of the females in UKB is 56.35 years old (std 8.00). We retrospectively analyzed personalized clinical information on diagnosis, medical procedures, lifestyle, personal genetics, self-reporting, and nurse interview reports. Following strict filtration steps (see Methods), we analyzed 148,571 women, among whom 5924 were diagnosed with endometriosis (ICD-10: N80, Data field).

Table 1 lists a selected sample of the different data types (e.g., physical measurement) that were used in this study. The extracted UKB fields cover information that is binary, contentious, or divided into discrete categories. The data were obtained from the participants’ medical records or by questionnaires and exams at assessment centers. Despite the effort to standardize and fill all data fields in UKB (Supplementary Table S1), some attributes and measurements suffer from a substantial fraction of missingness. For example, while only 2.7% of the female population of this study (148.5k) lacked menarche age, the ages of the first and last age of depression episodes were missing for 78.5% of the participants that experienced depression.

In summary, the data extraction following the filtration scheme covered 970 ICD-10 diagnoses, two sets of genetic variants (with 65 or 399 variants), and 46 attributes from lifestyle and physical measures. The extraction of data was motivated by endometriosis risk factors previously studied and expanded according to input from medical experts.

### 3.2. Univariate Statistics of Control and Endometriosis Patients from the UKB

**Figure 1:**
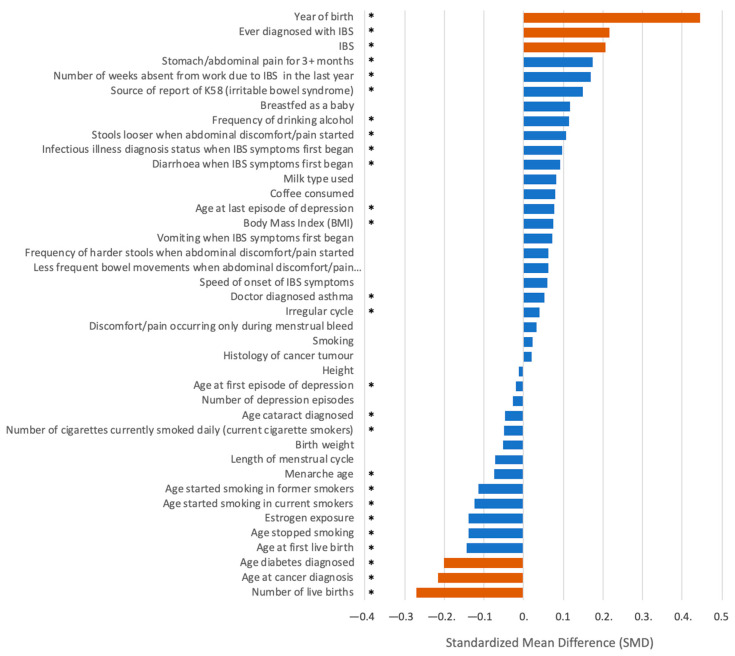
Univariate analysis for endometriosis. A ranked list of attributes (total of 44) associated with endometriosis diagnosis and control groups by the standardized mean difference (SMD). SMD values <−0.2 and >0.2 are colored orange to indicate those with a substantial effect size. The statistics were based on the median calculated for the Q1–Q3 values. An asterisk (*) next to the description of the attribute is the case with a *p*-value <0.05 for univariate tests of cases and controls (see Methods). For a univariate statistical test and results, see Supplementary Table S1.

A post hoc statistical test was performed to assess the contribution of each individual measurement. Numerous attributes have been previously reported as risk factors for endometriosis. Figure 1 shows the differences between the endometriosis group and the control group based on SMD (see Methods). Each attribute was independently analyzed by including the median values (Q1, Q3) and calculating the statistical significance of its effect size. Setting the SMD threshold at 0.2, only six (out of 44) attributes were strongly associated with risk for endometriosis. The number of live births and the age at cancer and diabetes diagnosis (UKB fields of 2734 and 40,008, respectively) suggest a lower risk for endometriosis. The most significant variable in accordance with an increased risk of endometriosis is the year of birth (SMD of 0.44) followed by irritable bowel syndrome (IBS). The rest of the measurements had smaller effect sizes. For detailed information, see Supplementary Table S1.

The calculated effect sizes associated with most of the attributes that were previously linked to endometriosis (e.g., menarche age, BMI, height, birth weight) were low. Other attributes failed to meet statistical significance (e.g., smoking, height, coffee consumed). Importantly, the factors in the ranked list shown in Figure 1 only partially overlap with the known risk factors for endometriosis as reported in the literature.

Assessing the risk according to the contribution of each attribute independently of the others cannot capture the non-additive interaction of specific factors. A likely scenario is that different factors (each carrying a marginal effect) interact, and their combination provides valuable predicting power. Moreover, extracted and engineered features may be of multiple types. Specifically, while some attributes are continuous (e.g., BMI), others are binary (e.g., having a specific ICD-10), and many are assigned by a few categories (e.g., smoking habits). Thus, we sought a method that considers any variable irrespective of its type.

**Figure 2:**
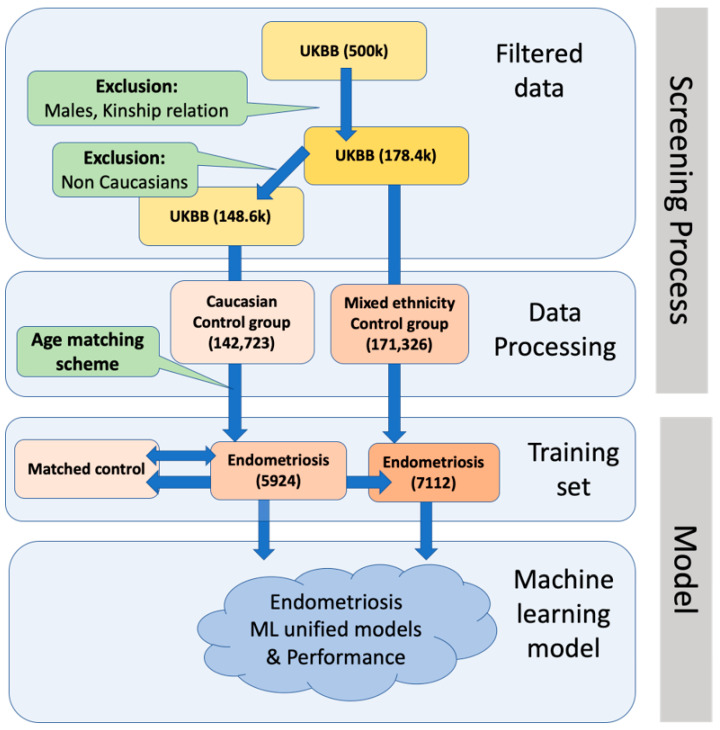
A scheme of data extraction, processing and machine learning models for endometriosis for the Caucasian and the mixed ethnicity populations.

For the goal of developing a predictive model for endometriosis, we applied a multivariate machine learning-based framework. A scheme of the analyses and processes for creating a predictive model for endometriosis using the UKB data is shown in Figure 2. In brief, following filtration, a screening process was applied, resulting in 148,571 participants, out of whom 5924 were diagnosed with endometriosis. The data were divided into two sets, 80% for training and 20% for testing. Each model was trained 10 times, while keeping the 80:20 ratio for the train and test sets. We further analyzed the data and its distribution to account for internal year-dependent biases (Figure 2, Data processing). By excluding males and kinship relations, we derived a mixed population of subjects of all ethnic origins (a total of 178,438), out of whom 7112 were diagnosed with endometriosis. This population is referred to as a “mixed ethnicity” population (see Methods). The calculated prevalence of endometriosis in the European origin cohort (145,671) and the mixed ethnicity populations in UKB is identical (3.99%). Note that the mixed population includes an addition of ~30,000 women, with ~1200 among them diagnosed with endometriosis (Figure 2). As genetic analyses perform best in populations with shared genetic origins, the unification of models with genetic input was limited to Caucasians (i.e., European origin cohort).

**Figure 3:**
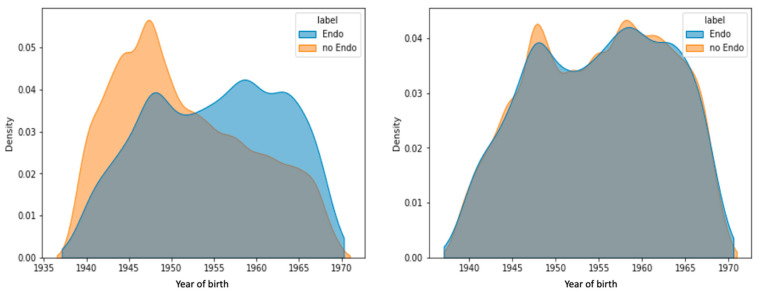
The distribution of the control and endo groups by birth year (Left). Following a protocol for yearly matching schemes, the bias was removed. Additionally, each year a matched proportion of control and endo-groups remained stable throughout (for a detailed protocol, see Supplementary Text S1).

Figure 3 shows the distribution of the participants in the study for women that were not diagnosed (control group) and those diagnosed with endometriosis (endo group). There was a significant difference in the year of birth distribution among women with and without endometriosis (U-test, *p*-value 2.2 × 10^−239^). To overcome this bias, we created a matched set for each year to cancel out the original year of birth differences. Repeating the U-test after applying the matching protocol resulted in an insignificant difference between the control group and the endo group. The rest of the analysis was performed on the age-matched data.

### 3.3. Predictive Risk Model for Endometriosis

After a screening process (Figure 2), the data were separated into three main categories according to the type of data used for training. These categories provided the basis for three models. We labelled the inputs a, b and c according to the type of data used, which were as follows (see Methods): (a) attributes and measurements from UKB (Figure 1, Supplementary Table S1); (b) medical diagnoses, as indexed by ICD-10 codes (Supplementary Table S3); and (c) genetic variants based on endometriosis GWAS (Supplementary Table S2).

**Figure 4:**
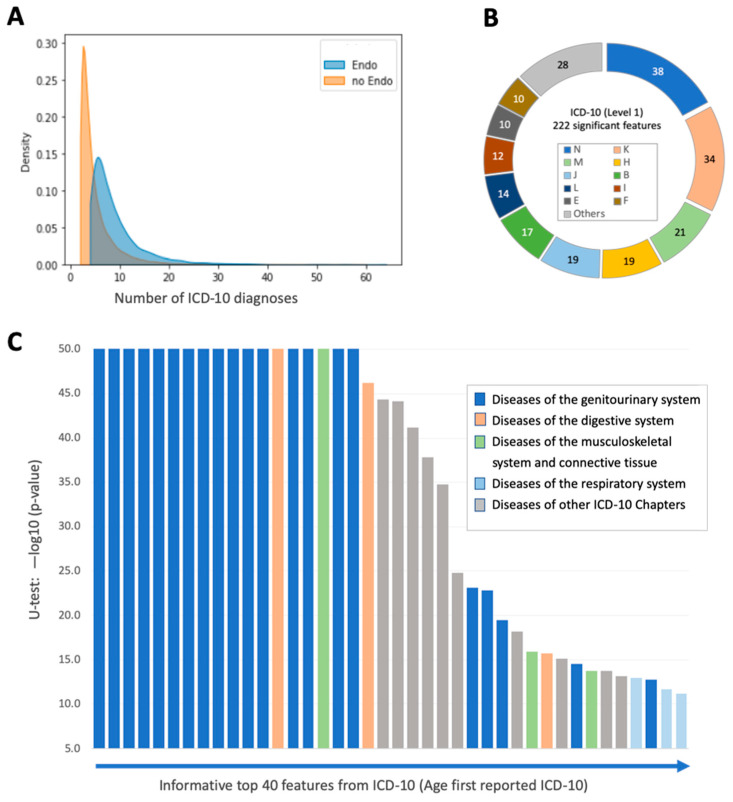
ICD-10 in control and endo-groups. (**A**) The distribution of the number of ICD-10 diagnoses in the control and endo-groups (orange and blue, respectively) was significant using the Mann-Whitney U-test (*p*-value < 0.001) and SMD = 0.471. The median value of the number of ICD-10 diagnoses per individual for the control and endo-groups is 1 and 4, respectively. (**B**) Partition of all 222 statistically significant informative features from the ICD-10 based model (U-test, *p*-value < 0.05). Each feature was tested for the statistical difference between the control and the endo-group. The partition is according to the ICD-10 level 1 first letter (A-Q). The level 1 letters with less than 10 features are unified (‘others’). (**C**) Ranked list of the top 40 ICD-10 that statistically differentiate ranked by the *p*-value < 1 × 10^−11^. These 40 ICD-10 codes are color coded as in B by level 1 ICD-10 index. Detailed information on the listed features and ICD-10 level 4 information is available in Supplementary Table S3.

In preparation for the data for model b (medical diagnoses), we collected the ICD-10 reported for each woman (i.e., a vector of ICD-10 diagnoses). Importantly, the UKB data fields provide the dates of the participants’ initial appearance of any medical diagnosis. These dates were converted into the age of the diagnosis for each woman (Supplementary Figure S2). The mean age of ICD-10 diagnosis of endometriosis (N80) is 42.1 (std = 10.6) years. Each diagnosis was assigned to the timeline of the individual age. Based on this protocol, we were able to define the set of ICD-10 diagnoses that preceded the date of endometriosis diagnosis, and removed any of the medical conditions and diagnoses that occurred after the alignment date. We attempted to find statistical differences in the amount and nature of the ICD-10 terms between cases and controls for use as informative features for endometriosis prediction. The rationale was to assess whether other diagnoses preceding the definitive endometriosis diagnosis carry a predictive power towards endometriosis. For each participant in the control group, a threshold age for the diagnosis masking was randomly chosen from the endometriosis diagnosis age, such that the threshold distribution in the control group was equal to the distribution of endometriosis diagnosis age. The median number of diagnoses prior to that of endometriosis for the controls and endo-group was 1 and 4, respectively (Figure 4A).

Supplementary Table S3 shows the percentage of ICD-10 terms associated with women with and without endometriosis for 755 age-associated diagnoses (see Methods). While only 7% of the control group had >10 ICD-10 diagnoses, as many as 11% of the endo group had more than 30 ICD-10 diagnoses. Each age-converted ICD-10 was tested for the statistical difference between the control and the endo-group. For 222 items, the “age of first reported diagnosis” resulted in *p*-value < 0.05 in a non-parametric statistical test (Supplementary Table S3). Figure 4B shows the partition of these 222 items according to the ICD-10 indexing method (level 1; marked A to Q; Supplementary Table S4). The abundant ICD-10 level 1 includes diseases of the genitourinary system (N), followed by diseases of the digestive system (K), diseases of the musculoskeletal system and connective tissue (M) and diseases of the respiratory system (J). The significance of diseases of the respiratory system (J) and viral and parasite infection (B) is less evident.

Figure 4C shows a ranked list of the most significant ICD-10 items according to U-test statistical results with pelvic and genital organs that prevail. Specifically, the most significant ICD-10 items included N73 (pelvic inflammatory diseases), N81 (female genital prolapse), noninflammatory disorders of the ovary, fallopian tube, and broad ligament (N83) and of the uterus, except cervix (N85), polyps of the female genital tract (N84), and excessive, frequent, and irregular menstruation (N92). Endometriosis knowledge confirms the importance of diseases associated with N, K, and M, as well as, to a lesser extent, diseases of the respiratory system (J).

In preparation for the machine learning predictive model, careful treatment of the data is required. For the genetic model (model c), we collected variants from GWAS of endometriosis as an input for the predictive model. A list of 65 genetic variants associated with 35 different genes was compiled from 11 major publications, including large meta analyses (17,045 endometriosis cases and 191,596 controls) [28]. The list was compiled from the OT genetic platform (Supplementary Table S2).

**Figure 5:**
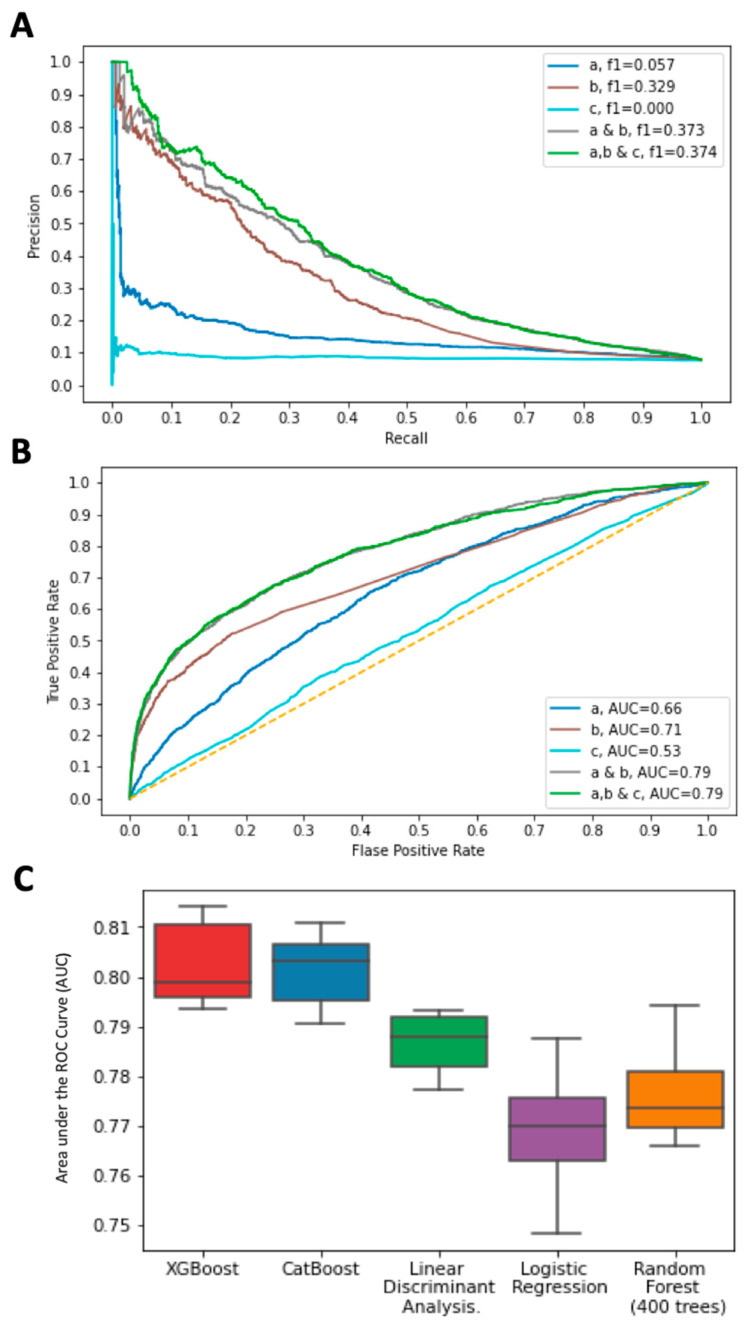
Performances of the prediction models for endometriosis. (**A**) Precision–recall curves for 5 CatBoost models. The models differ by training data with the UKB attributes and measurements (model a), the collection of the ICD-10 prior to endometriosis diagnosis age (model b), and the GWAS of endometriosis genetic variants (model c). A combination of training data of a and b and a combined model that includes a, b and c. (**B**) ROC curves for the same set of 5 models as in A. The diagonal line marks a random no-discrimination line (AUC = 0.5). (**C**) A comparison of the ROC-AUC of five different algorithms for the combined set of input features a, b, and c. XGBoost and CatBoost resulted in the highest performance according to ROC-AUCs.

Figure 5 shows the results from the performance by ROC-AUC for five models based on the major data type categories (marked a, b, and c; see Methods (Section 2)) and their combinations. The predictive models for each of the data types (a–c) and their combinations are shown for the combination of recall and precision in Figure 5A and the ROC-AUC is presented of all five models in Figure 5B. Developing a model based on the 65 variants from the GWAS catalog (model c) indicated that training the model on genotypic data resulted in an ROC-AUC of 0.53 (where 0.5 suggests no discrimination). A recent population-based polygenic risk score (PRS) analysis for endometriosis showed only 2–3% of the variance explained by the SNPs [37], consistent with the modest improvement in the performance of model c. We therefore tested whether expanding the list of associated variants from GWAS by including those with a lower significant threshold carried discriminative information in the case–control setting. To this end, we created an endometriosis–PRS model with 399 variants [37]. A non-parametric U-test was used to compare the PRS for endometriosis for the control and endo groups (87,080 and 4354, respectively). The analysis confirmed that the two cohorts display no difference (*p*-value = 0.172; SMD = 0.02). Supplementary Figure S3 lists the 399 variants used for the PRS of endometriosis [37].

We found that model c (GWAS variants), in combination with model a (measurements and attributes from self-reporting and lifestyle data) and model b (age-converted ICD-10 diagnoses prior to endometriosis), resulted in an ROC-AUC that is identical to that of a combined model of a and b (0.79, Figure 5B). We concluded that the contribution of the genetic effect from GWAS results is negligible (Supplementary Table S5).

We repeated training with inputs a, b and c to test the performance of additional machine learning models (Figure 5C). The results of the models performed by Random Forest, Logistic regression, Linear discriminant analysis, XGBoost, and CatBoost algorithms are shown. The CatBoost algorithm of the combined model outperformed other models, followed by XGBoost (Supplementary Table S6). The AUC, which was associated with additional algorithms including K-nearest neighbors (KNN), Naive Bayes (NB), and support vector machines (SVM), resulted in poor performance (not shown).

Repeating the training of the model with input from the UKB mixed ethnicity population for models a and b resulted in the same results as obtained for the Caucasian population, supporting the notion that the ICD-10 diagnoses and variables of lifestyle and physical measurement are robust and valid for mixed ethnicity.

### 3.4. Informative Features and Interpretability of the Combined Model

**Figure 6:**
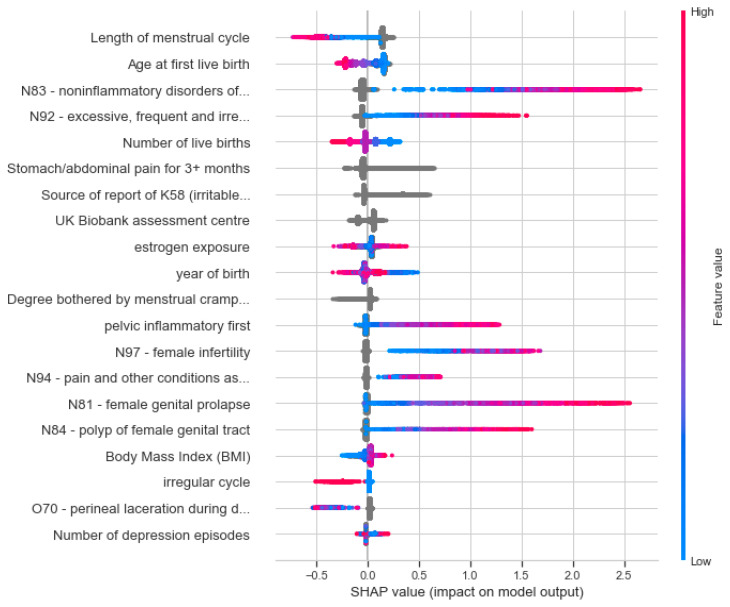
Top 20 features from the combined model using SHAP (an explainable AI tool). Variables are ranked in descending order of their SHAP value, where the most informative feature is at the top. The values reported show the contribution of each of the features according to the impact of that feature on the model outcome (i.e., endometriosis). Each dot in the plot represents a subject patient’s feature value for that variable (vertical axis). Color reflects the scale of the feature’s value. Color shows whether that variable is high (red) or low (blue) for that observation. Gray depicts no data or a categorical feature.

We further evaluated the contribution of each feature to the combined model that was trained on three groups of features (a, b, and c) using SHAP, an explainable AI tool. Figure 6 shows the top 20 features ranked by SHAP. About a third of these features are associated with features of the age-dependent ICD-10, level 1 (Figure 4B), with the rest derived from the features associated with measurements and UKB attributes. The top features are the length of the menstrual cycle and the age of the first live birth. Note that none of the genetic variants (from GWAS variant lists of 65 or 399) were selected to be among the most informative 20 features. Figure 6 also emphasizes the limited overlap between SHAP informative features and the attributes with significant SMD from the univariate test (Figure 1).

The significant SHAP values support the contribution of noninflammatory disorders of the ovary, fallopian tube, and broad ligament (SHAP value of 0.134), and excessive, frequent, and irregular menstruation (N-92, SHAP value of 0.124). The informative features ranked by SHAP (e.g., estrogen exposure, reports of IBS) also displayed a strong deviation in occurrence in the endo group and control groups. However, statistically significant features from the ICD-10 diagnoses by age are abundant in the endo group relative to the control group, not selected as informative features by SHAP. This list includes the age of the first occurrence of N39 (other disorders of the urinary system), I10 (essential, primary, hypertension) and D50 (iron deficiency anemia) with *p*-values of 7 × 10^−55^, 6 × 10^−42^, and 2 × 10^−35^, respectively.

**Figure 7:**
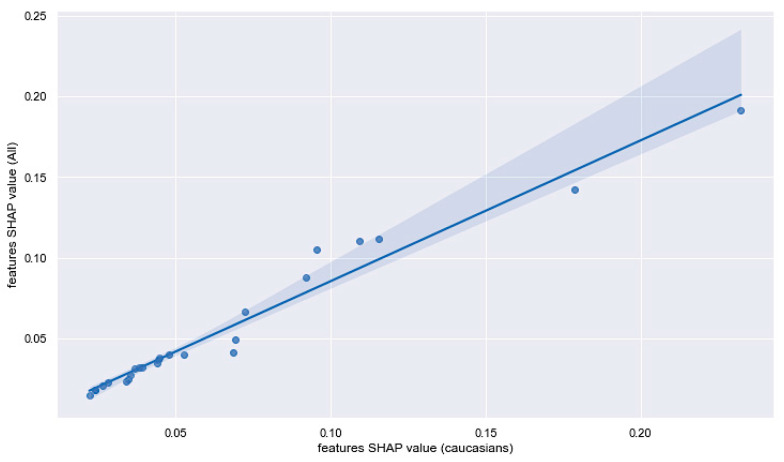
Scatter plot for the top 20 features from the model (for input a and b, excluding genetics) using SHAP. Each dot in the plot represents the feature value for a variable for the Caucasians and the mixed ethnicity populations. The shaded area marks the 95% confidence interval.

We compared the rank and the SHAP values of the models (a and b) using the population with mixed ethnicities relative to the Caucasian women’s cohort. Figure 7 shows the Pearson correlation for the top 20 features selected by their SHAP values in the two sets. While a few of the SHAP values deviated from the 95% confidence interval, the order of the selected features remained identical for the two tested sub-populations. Supplementary Table S7 lists the top 100 selected features along with their SHAP values for the Caucasian and mixed ethnicity populations.

### 3.5. Model’s Limitation

Almost all the analyzed data used for our models were based on measurements observed in women after their menopause age. Thus, the most up-to-date diagnostic measurements were unavailable. The presented models (Figure 5C) were not designed as tools for diagnosis. However, we engineered features that include information collected prior to the date of diagnosis of endometriosis (i.e., to avoid complications and outcomes that occur years after a definitive endometriosis diagnosis). Due to a lack of awareness during the relevant years in the 20th century (Figure 3), the prevalence of endometriosis-affected women in our cohort is 4%, which is slightly lower than current estimates (5–10%). While all women recruited were in the age range of 40–69, the age of a definitive diagnosis was recorded (Supplementary Figure S2). The average age of endometriosis diagnosis is 42.1 years (Q1 and Q3 are associated with 35 and 49 years of age, respectively). Considering the delay in the definitive diagnosis of ICD-10 N80 from the onset of symptoms (7–11 years), we confirmed that most women were diagnosed during their reproductive years. Another limitation of this study concerns the long time to diagnosis. This may cause uncertainty in partition diseases before and after endometriosis diagnosis (i.e., endometriosis alignment date). Another aspect that may limit the generality of our model concerns an unavoidable enrichment in women with symptomatic or severe endometriosis. We anticipate that data analyzed from these women may not represent mild manifestations of endometriosis. In terms of UKB data quality, for data fields of UKB diagnosis that lack a timestamp, it could not be determined whether they occurred before endometriosis diagnosis.

## 4. Discussion

The goal of this study was to explore endometriosis risk factors by developing a predictive model based on population-based data. With the increased availability of biobanks (e.g., UKB) and rich individual medical and genetic data, the development of a reliable and robust model for endometriosis is of utmost importance. In practice, information on the number, location, and size of the lesions does not correlate with the patient’s pain severity, fertility, or therapy success [41]. Researchers can use predictive risk models to better understand the etiology and underlying mechanisms of endometriosis [32,42].

The current lack of an effective diagnosis of endometriosis leads to delayed or missed diagnosis with an average latency of 7–11 years from the onset of symptoms to definitive diagnosis [7]. These years prior to diagnosis are associated with reduced quality of life [43] and high financial costs to the patient and the healthcare system. In addition, experiencing recurrent pain often impacts one’s psychological and mental state, leading to a substantially compromised quality of life [21]. Early diagnosis may impact future health in several ways [44], such as in the case of the malignant transformation of ovarian endometriomas into ovarian cancer [45,46]. Importantly, endometriosis is a chronic inflammatory disease that can progress. With an early diagnosis, the appropriate medical treatment can be prescribed, avoiding the progression of the disease and its consequences (i.e., chronic pelvic pain, infertility, surgeries) [7,12,16]. Despite extensive efforts to identify biomarkers (e.g., miRNA, peptides, metabolites) and to establish non-invasive indicators [47], diagnostic tests based on biomarkers from peripheral blood have not been validated [48]. In this respect, screening for biochemical indicators can benefit from the growth in population-based body fluid biobanks (e.g., blood, urine) [48]. Recently, a scoring system was developed and validated based on a detailed endometriosis-related questionnaire. The clinical application of such a scoring method (refined to a small number of informative items) was proposed as a cost-effective approach to reduce diagnosis delays and improve quality of life [49].

Our model emphasizes the utility of population-based data resources such as the UKB for studying endometriosis. As the recruitment of participants to the UKB is not disease specific, the studied groups are expected to be relatively resistant to selection bias. Nonetheless, the data in the UKB are not ideal for studying endometriosis, mainly because a large fraction of the women have reached postmenopausal age [50]. We addressed these difficulties by carefully preprocessing and matching the data. It is anticipated that a bias by the year of birth for the endo group is probably a reflection of establishing the diagnosis protocol and a change in the diagnosis rate (Figure 3). This is probably also due to an increase in awareness, and the introduction of medical procedures for definitive diagnosis [7]. We implemented an age-matching protocol to secure the age-balance of the studied groups. Another concern is the use of ICD-10 diagnoses. As a predictive risk model, we aligned each ICD-10 item with respect to endometriosis by converting the data of the first disease occurrence to the women’s age. We ignored all diagnoses that were dated after endometriosis was diagnosed. In our model, we did not include any molecular measurements (e.g., miRNAs from biopsies, drug use) [51]. Instead, we included data fields from electronic health records (EHR) to develop reliable predictive models. Menarche age, smoking, and BMI were not proposed as strong indicators of endometriosis in any of our endometriosis models (Figure 6). We believe that it is fundamental to revisit potential risk factors and assess their relevance to clinical recommendations and disease diagnosis.

From a clinical perspective, our study confirmed the associations with diseases of the genitourinary system (N), the digestive system (K), and diseases of the musculoskeletal system and connective tissue. Irritable bowel syndrome (IBS) was identified as an informative feature in many of the models. A recent meta-analysis provided epidemiological evidence for a link between IBS and endometriosis [52]. It shows that there is a higher risk (>2 fold) of IBS in women with endometriosis compared to women without the condition [53]. However, the occurrence of other diseases, such as migraine (G43) and dorsalgia (M54) in a substantial fraction of the women within the endo group (>5%) was less evident. A large genetic meta-analysis to identify the shared genetic basis of endometriosis and other diseases identified dorsalgia as having a significant positive genetic correlation with endometriosis [54]. It was further shown that a sensitivity to pain might be shared by other pain-associated diseases. The feature “stomach pain for 3 or more months” was ranked high in the final model (Figure 6). This information was collected only from participants who indicated that in the last month they experienced stomach or abdominal pain. The possibility that stomach pain in post-menopausal years echoes the prolonged pain experienced during fertile years should be tested in an independent cohort. The co-occurrence of endometriosis with other diseases such as asthma (J45) and iron-deficiency anemia (D50) may reflect missed or overdiagnosis prior to the definitive diagnosis of endometriosis.

The effect associated with genetic variants in complex diseases and traits might be rather limited and strongly influenced by the amount of variation due to genetic factors (i.e., heritability). The polygenic risk scores (PRS) for endometriosis rely on the summarizing effects of GWAS studies [55]. In this study, we included 65 variants associated with 35 genes from the harmonized collection of GWAS (Supplementary Table S2). Several of these variants were validated across populations (e.g., Japanese descent and European cohorts [56]). Endometriosis PRS revealed that the GWAS variants explained only 2–3% of the phenotypic variance [57,58], arguing for insufficient clinical utility. The PRS developed using an extended list with 399 variants failed to distinguish between the control and endo groups (Supplementary Figure S3). In our machine learning framework, the variants slightly contributed to the discriminatory value (Figure 5B). It emphasized the benefit of including not only genetic signals, but also orthogonal medical and environmental data into a single model, as exemplified for Type 2 diabetes (T2D) [59]).

The performance of machine learning models is usually evaluated by the observed accuracy, F1-score, and ROC-AUC. However, robust and reliable models must show resistance to data leakage, a term that stands for the ability of the algorithm to learn a simple value for ‘trivial’ discrimination. During our study, we realized that our model showed great sensitivity towards such (explainable and hidden) leakages. Data leakage carries the risk of achieving almost perfect performance on a dataset while lacking generalizability in the real world. For example, a feature that led to a leak was “estrogen exposure”. Inspection revealed that the model learned to identify the exceptionally short “estrogen exposure” years. It is an outcome of hysterectomies, which was associated with endometriosis treatment [60]. A similar leakage was attributed to the “age at last live birth”. A model using these “leaky” features would predict endometriosis with an outstanding AUC score of 0.94. We reduced the model leakages by adjusting the parameter distributions between the endo and control groups. In cases where such an adjustment was insufficient, we removed features (e.g., age of last birth).

With the increasing use of medical imaging, videos, and pathological samples, machine learning and deep learning approaches are playing a growing role in diagnosis [61]. A machine learning model for endometriosis based on a screening questionnaire was shown to produce an AUC of 0.5–0.9 in the training and validation sets based on the combination of 16 common criteria such as age, pain, and family history [62]. We demonstrated that the reanalysis of large cohorts of diagnosed women with endometriosis from the general population of UKB provided attributes and measurements not traditionally associated with the disease, and which were not informative under standard univariate statistical tests. Moreover, we confirmed that the model is generalized and its performance remained identical between European ancestry and mixed ethnicity populations (20% increase in cohort size). It is anticipated that the incorporation of explainable models into the clinics will have an impact on the personalized approach and will lead to a reduction in the latency in endometriosis diagnosis.

## Figures and Tables

**Table 1:** Sample of extracted data fields from UKB used in this study.

Attributes & Traits (Units)	Data Type Class	UKB Field ID	Number of Women	Missing Data (%)	Mean [Cardinality]
Body mass index (BMI)	Physical measures	21001	148,026	<1	27.2
Smoking	Lifestyle & environment	20116	37,444	74.8	[4]
Birth weight (kg)	Early life factors	20022	52,645	35.5	3.32
Number of live birth	Female-specific factors	2734	148,402	<1	1.8

## Data Availability

Not applicable.

## Supplementary Materials

The following supporting information can be downloaded at: https://www.mdpi.com/article/10.3390/jpm12071114/s1, Text S1: Pseudocode for age alignment for control and endo groups; Figure S1. Missing data in variables from UKB. Figure S2. The distribution of age of endometriosis diagnosis. Figure S3. Endometriosis-PRS of 399 variants. Table S1: Measurements and attributes from UKB and univariable statistics [Source for Figure 1]. Table S2: GWAS of endometriosis, variants extracted from OT genetic platform. Table S3: Features extracted from ICD-10 and statistics of endo group vs. control group [Source for Figure 4]. Table S4: Number of statistically significant associated features linked to the chapters of ICD-10, level 1 [Source for Figure 4]. Table S5: Performance of predictive models for endometriosis using CatBoost [Source for Figure 5]. Table S6: Comparing machine learning algorithms for combined models (10 iterations each) [Source for Figure 5C]. Table S7: Informative features from the combined model, ranked by SHAP for Caucasians and mixed ethnicity populations.

## Abbreviations

AI	Artificial Intelligence
AUC	Area Under the ROC Curve
DL	Deep Learning
HER	Electronic Health Records
OT	Open Targets
ROC	Receiver Operating Characteristic Curve
IBS	Irritable bowel syndrome
UKB	UK-Biobank
PRS	Polygenic Risk Score
T2D	Type 2 Diabetes
BMI	Body Mass Index
