# Supplementary material for: Revisiting the Risk Factors for Endometriosis: A Machine Learning Approach
Source: J Pers Med. 2022 Jul 7;12(7):1114. doi: 10.3390/jpm12071114 (PMC9317820; doi:10.3390/jpm12071114)
Supplement: Supplementary file 1 [file jpm-12-01114-s001.zip › ENDO-supplelemtnal -Cyc-4-TextS1+Fig-S1-S3+Tables S4-S6.pdf]

Revisiting the Risk Factors for Endometriosis: A Machine Learning Approach

Ido Blass[1], Tali Sahar[2], Adi Shraibman[3], Dan Ofer[5], Nadav Rappoport[4], Michal Linial[5*]

Supplementary Data

Text S1. Pseudocode for age-matching of endometriosis diagnosed and control groups

Algorithm 1 same year of birth distribution training set

$\mathcal{D} \leftarrow$ The data set after screening process

$\mathcal{Y} \leftarrow \{y|$ A woman in $\mathcal{D}$ was born in the year $y\}$ ▷ Set of years

$\mathcal{N}_y \leftarrow \{s \in \mathcal{D}|s$ was not diagnosed with endometriosis & born in the year $y\}$

$h^e_y \leftarrow$ number of women which were diagnosed with endometriosis & born in the year y

$h^{ne}_y \leftarrow$ number of women which were not diagnosed with endometriosis & born in the year y

$\alpha \leftarrow \max_n \{n \in \mathbb{N} | \forall y \in \mathcal{Y}\ h^{ne}_y - h^e_y \cdot n \geq 0\}$ ▷ $\forall y \in \mathcal{Y}\ \ v^{n-endo}_y >> v^{endo}_y$

$\mathcal{T} \leftarrow \emptyset$

for $y \in \mathcal{Y}$ **do**

  $\mathcal{T} \leftarrow \mathcal{T} \bigcup \{$randomly chosen $\alpha \cdot v^{endo}_y$ samples from $\mathcal{N}_y\}$

end for

$\mathcal{T} \leftarrow \mathcal{T} \bigcup \{$s $\in \mathcal{D}|$s diagnosed with endometriosis$\}$ ▷ Training data set

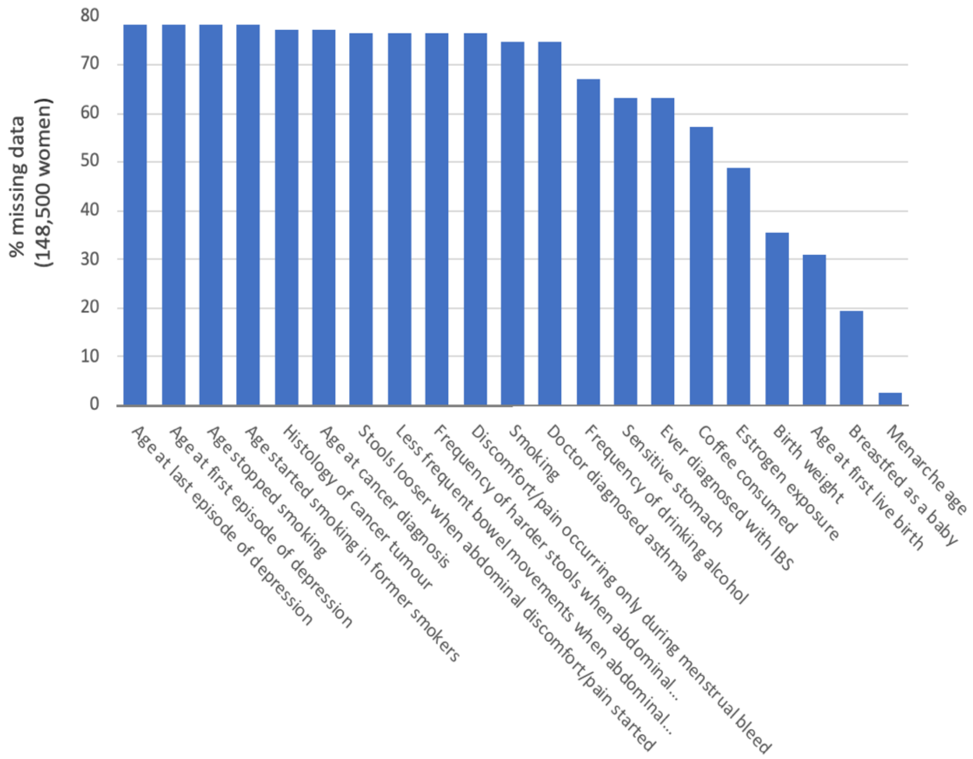

Figure S1. Ranked list of variables with the percentage of the missing data. The data covers the attributes with 2% to 80% missing data. A full list of extracted attributes is available in Supplementary **Table S1**.

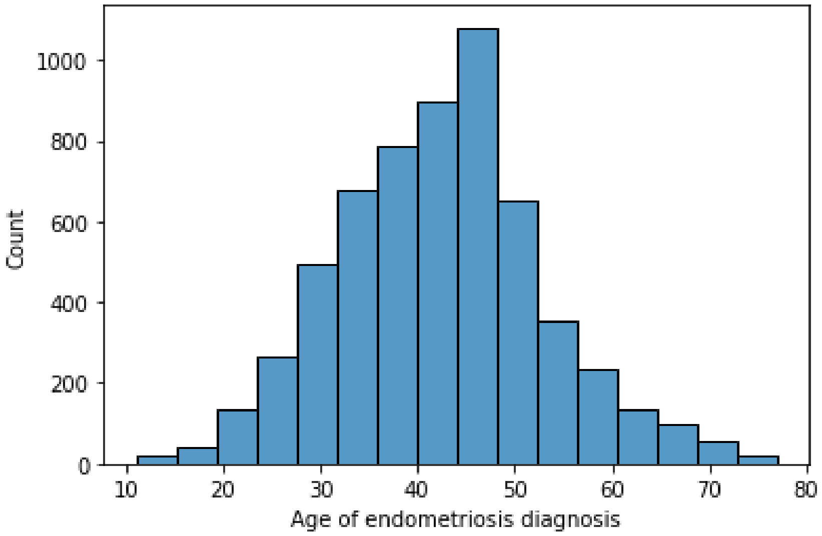

Figure S2. Age distribution of ICD-10 N80 in the UKB. The average age is 42.12 (std = 10.61) years The Q1 (25%) and Q3 (75%) are 35 and 49 years, respectively.

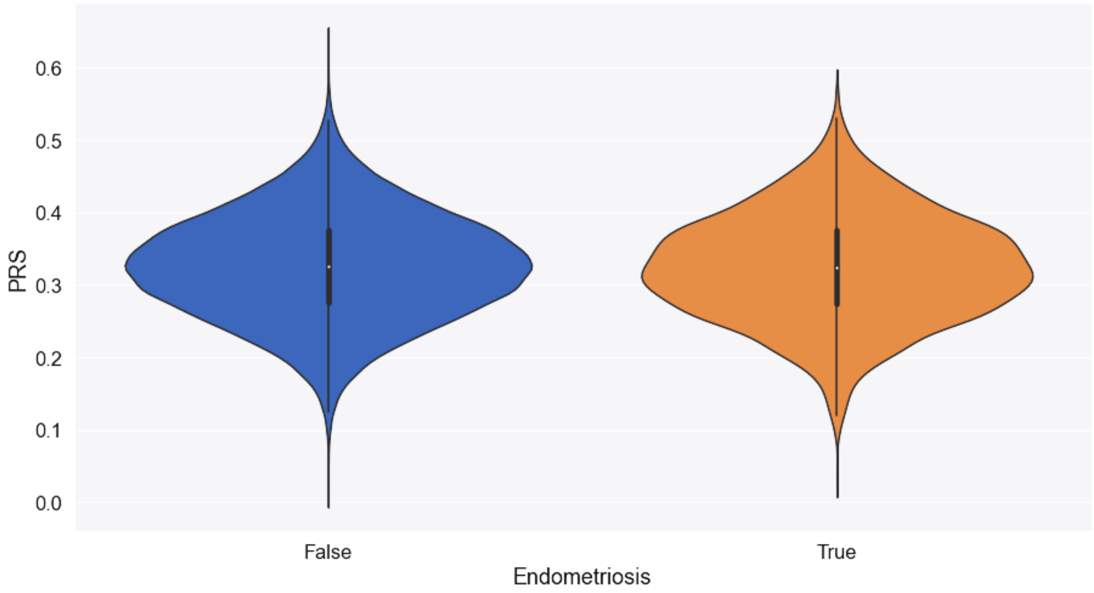

Figure S3. Violin plot of control and endo groups by creating a PRS based on 399 variants (Supplementary **Table S2)** extracted from GWAS from UKB and Ireland (Prive et al, 2022). The U-test comparing control group (87,080) and endo group (4,354) resulted with p-value = 0.172; SMD = 0.02, confirming no difference in the distribution of the two groups.

Table S4. Chapters of ICD-10 level 1, and number of statistically significant associated features

ICD-10 level 1[a]	Chapter (level 0)	# of features (total 222)
N	Chapter XIV: Diseases of the genitourinary system	38
K	Chapter XI: Diseases of the digestive system	34
M	Chapter XIII: Diseases of the musculoskeletal system and connective tissue	21
H	Chapter VII: Diseases of the eye and adnexa	19
J	Chapter X: Diseases of the respiratory system	19
B	Chapter I: Certain infectious and parasitic diseases	17
L	Chapter XII: Diseases of the skin and subcutaneous tissue	14
I	Chapter IX: Diseases of the circulatory system	12
E	Chapter IV: Endocrine, nutritional and metabolic diseases	10
F	Chapter V: Mental and behavioural disorders	10
Others		28

[a]Others include all ICD-10 chapters with <10 features each.

Table S5. Performance of predictive models for endometriosis using CatBoost

Model	F1-score	Precision	Recall	Accuracy	ROC-AUC
a	0.057	0.383	0.031	0.921	0.663
b	0.329	0.472	0.253	0.920	0.718
c	0.000	0.000	0.000	0.922	0.522
a & b	0.373	0.499	0.298	0.922	0.784
a, b & c	0.374	0.502	0.298	0.923	0.784

Table S6. Comparing machine learning algorithms for combined models (10 iterations each)

Iterations	XGB	CB	LDA	LR	RF (400)
iteration 0	0.808	0.804	0.793	0.776	0.794
iteration 1	0.796	0.795	0.777	0.761	0.772
iteration 2	0.795	0.790	0.782	0.782	0.766
iteration 3	0.800	0.797	0.793	0.768	0.767
iteration 4	0.797	0.804	0.789	0.768	0.770
iteration 5	0.813	0.811	0.783	0.772	0.779
iteration 6	0.797	0.803	0.780	0.758	0.770
iteration 7	0.814	0.807	0.789	0.776	0.787
iteration 8	0.793	0.795	0.787	0.748	0.782
iteration 9	0.811	0.810	0.793	0.788	0.775
**mean**	**0.802**	**0.802**	**0.787**	**0.770**	**0.776**
std	0.008	0.007	0.006	0.011	0.009
